# A new record of critically endangered *Saussurea bogedaensis* (Asteraceae) from Dzungarian Gobi, Mongolia

**DOI:** 10.3897/phytokeys.160.55603

**Published:** 2020-09-08

**Authors:** Shukherdorj Baasanmunkh, Nudkhuu Nyamgerel, Gun-Aajav Bayarmaa, Batlai Oyuntsetseg, Khurelpurev Oyundelger, Hyeok Jae Choi

**Affiliations:** 1 Department of Biology and Chemistry, Changwon National University, Changwon 51140, South Korea Changwon National University Changwon South Korea; 2 Department of Biology, School of Arts and Science, National University of Mongolia, Ulaanbaatar 14201, Mongolia National University of Mongolia Ulaanbaatar Mongolia; 3 Department of Botany, Senckenberg Museum of Natural History Görlitz, D-02826, Görlitz, Germany Senckenberg Museum of Natural History Görlitz Germany

**Keywords:** Asteraceae, conservation status, distribution, Mongolia, *
Saussurea
*

## Abstract

A species in the family Asteraceae, *Saussurea bogedaensis*, was newly described from Bogeda Mountain in Xinjiang, China and is a critically endangered species in China. Morphological and genetic characteristics confirm the presence of this species in Mongolia, as it was found in Baitag Bogd Mountain (in the Dzungarian Gobi). In addition, the distribution and conservation status of *S. bogedaensis* are provided.

## Introduction

*Saussurea* DC. is one of the largest genera in the tribe Cardueae (Asteraceae) and comprises ~500 species, classified into six subgenera and 20 sections (Lipschitz 1979; Raab-Straube 2017). The genus is distributed throughout the Northern Hemisphere, with diverse species in Central Asia (Wang et al. 2009). *Saussurea* spp. occur in a wide range of habitats, especially at higher altitudes with cold and dry conditions, but they also grow in lowlands. However, *Saussurea* spp. have a tendency towards habitat specificity (Butola and Samant 2010). The highest number of *Saussurea* spp. is reported from China, with estimated 317 species (Chen and Yuan 2015). Sixty-one species are noted as native to India (Hajra 2000; Ahmad 2005), 54 species are mentioned in the flora of Siberia (Shurupova and Zverev 2017), 41 indigenous species are listed in the flora of Bhutan (Grierson and Springate 2001) and 23 species have been recorded in Pakistan (Ahmad 2005). To date, 53 species of *Saussurea* have been recorded in Mongolia (Gubanov 1996; Urgamal et al. 2014; Dariimaa 2017). Amongst these, five species, namely *S. catharinae* Lipsch., *S. gubanovii* Kamelin, *S. klementzii* Lipsch., *S. ramosa* Lipsch. and *S. saichanensis* Komarov ex Lipsch. are endemic to Mongolia (Urgamal and Oyuntsetseg 2017).

*Saussurea* is known for its wide array of uses, especially for medicinal and religious purposes (Mishra et al. 2018; Qureshi et al. 2018; Semwal and Painuli 2019). Additionally, the essential oils of several species are used in high-grade perfumes and as insecticides (Butola and Samant 2010). As a result of having a number of medicinal properties and economic uses, several *Saussurea* species are becoming threatened or endangered owing to over-exploitation and degradation of their habitats (Kamalpreet et al. 2019), as well as their natural rarity and small population size. These valued species include *S. involucrata* (Kar. & Kir.) Sch.Bip. and *S. orgaadayi* Khanm & Krasnob. which are listed as endangered species in the conservation list of Mongolia (Oyuntsetseg et al. 2018).

Our study initially aimed to clarify the taxonomic relationship between *S. involucrata* and *S. orgaadayi* in Mongolia and to assess the conservation status of these species. These two species are classified as endangered at the regional level and occur only in the western part of Mongolia (Grubov 1982; Gubanov 1996; Urgamal et al. 2014; Dariimaa 2017). Furthermore, both species have some morphological similarities, leading to misidentifications. Regarding their distribution range, *S. involucrata* is noted in four phytogeographical regions in western Mongolia: Mongolian Altai (MA), Dzungarian Gobi (DzG), Khovd and the Depression of Great Lakes (Grubov 1982; Dariimaa 2017). *Saussurea orgaadayi* is only noted in the MA region (Urgamal et al. 2014; Oyuntsetseg et al. 2017). *Saussurea involucrata* and *S. orgaadayi* belong to the Saussurea subg. Amphilaena, known for its taxonomic complexity (Raab-Straube 2017). However, *S. orgaadayi* can be differentiated from *S. involucrata* based on morphological characteristics of the capitula (Shi and Raab-Straube 2011; Raab-Straube 2017).

Recently, Chen and Wang (2018) discovered a new *Saussurea* species from Bogeda Mountain (Mt) in Xinjiang, China and named it *S. bogedaensis* Yu J.Wang & J.Chen. This newly-described species is closely related to *S. involucrata* and *S. orgaadayi*. This species had also been misidentified owing to its morphological similarity to *S. involucrata* and *S. orgaadayi*. Due to this confusion, Chen and Wang (2018) comprehensively investigated all three species and revealed some differences in their morphological characteristics, geographical distribution and phylogenetic positions. They also noted that *S. orgaadayi* was recorded in the Altai Mountains (Mts), and *S. involucrata* in the western part of the Chinese Tien-Shan Mts (Shi and Raab-Straube 2011; Chen and Wang 2018). *Saussurea involucrata* has been known to occur both in the DzG and MA regions of Mongolia (Urgamal et al. 2014). However, based on the distribution range indications of Chen and Wang (2018), the species recorded in the Mongolian MA is likely to be *S. orgaadayi*. Thus, inconsistencies in the distribution range of these three *Saussurea* species, which could have been misidentified in Mongolia as well, motivated us to conduct an in-depth taxonomic assessment. In addition, Chen et al. (2019) recommended the use of nuclear ribosomal (nr) DNA ITS and chloroplast (cp) DNA regions of *rbcL* and *trnH-psbA* as candidate DNA barcode markers for species in the subg. Amphilaena. Using these three markers, it was possible to discriminate the *Saussurea* species that are morphologically similar and separated very recently.

The main objectives of the present study were to (1) re-identify the above mentioned *Saussurea* species recorded in western Mongolia and (2) newly report *S. bogedaensis* and describe its distribution and conservation status in the Mongolian flora.

## Materials and methods

### Herbarium and field research

The basic distribution data and photographs of the target *Saussurea* species, which had been known as *S. involucrata* and *S. orgaadayi* in Mongolia, were collected during our fieldwork from 2013 to 2019 in western Mongolia. We also included herbarium materials kept at UBA, UBU, OSBU and MW (abbreviations are according to Thiers 2019+).

### DNA barcoding

In this study, we investigated the application of combined nrDNA region of ITS and cpDNA regions of *trnK*, *trnH-psbA* and *rbcL* in barcoding analyses of two Mongolian *Saussurea* species. Additionally, a total of 36 sequences, based on four markers of three species (*S. bogedaensis*, *S. orgaadayi* and *S. involucrata*), which were used by Chen and Wang (2018) to evaluate the phylogenetic relationships between these species, were obtained from NCBI GenBank (Table 1). *Jurinea multiflora* (L.) B.Fedtsch. was selected as an outgroup based on Chen and Wang (2018) and Chen et al. (2019). Detailed information on sample collection, voucher specimens, Genbank accession numbers and references of each sample is provided in Table 1.

**Table 1.** Detailed information on taxa, sampled locations, voucher specimens, NCBI GenBank accession numbers and references of the samples used in this study.

Taxon	Location & Herbarium accession number	Latitude (N) / Longitude (E)	Altitude (m)	GenBank accession number				Reference
				ITS	*rbcL*	*trnK*	*trnH-psbA*	
*S. bogedaensis*	Mongolia, Dzungarian Gobi; UBU20190698	45°13'14.52", 90°55'12.97"	2742	MT209829	MT624048	MT624054	MT624060	This study
*S. bogedaensis*	Mongolia, Dzungarian Gobi; UBU20190699	45°13'14.52", 90°55'12.97"	2742	MT210906	MT624049	MT624055	MT624061	This study
*S. bogedaensis*	Mongolia, Dzungarian Gobi; UBU20190700	45°13'14.52", 90°55'12.97"	2742	MT197331	MT624050	MT624056	MT624062	This study
*S. bogedaensis*	China, Xinjiang, Qitai; WYJ201308006 (38)	43°27'11.56", 89°33'7.67"	3471	MH003708	MH070873	MH070999	MH070746	Chen et al. (2019)
*S. bogedaensis*	China, Xinjiang, Qitai; WYJ201308006 (39)	43°27'11.56", 89°33'7.67"	3471	MH003709	MH070874	MH071000	MH070747	Chen et al. (2019)
*S. bogedaensis*	China, Xinjiang, Qitai; WYJ201308006 (40)	43°27'11.56", 89°33'7.67"	3471	MH003710	MH070875	MH071001	MH070748	Chen et al. (2019)
*S. orgaadayi*	Mongolia, Mongolian Altai; UBU20180340	46°51'08.6", 91°45'27.3"	2848	MT209870	MT624051	MT624057	MT624063	This study
*S. orgaadayi*	Mongolia, Mongolian Altai; UBU20180341	46°51'08.6", 91°45'27.3"	2848	MT209871	MT624052	MT624058	MT624064	This study
*S. orgaadayi*	Mongolia, Mongolian Altai; UBU20180342	46°51'08.6", 91°45'27.3"	2848	MT210907	MT624053	MT624059	MT624065	This study
*S. orgaadayi*	China, Xinjiang, Altai; WYJ201308041 (11)	47°13'6.46", 89°52'47.96"	3541	MH003773	MH070934	MH071060	MH070807	Chen et al. (2019)
*S. orgaadayi*	China, Xinjiang, Altai; WYJ201308041 (12)	47°13'6.46", 89°52'47.96"	3541	MH003774	MH070935	MH071061	MH070808	Chen et al. (2019)
*S. orgaadayi*	China, Xinjiang, Altai; WYJ201308041 (360)	47°13'6.46", 89°52'47.96"	3541	MH003775	MH070936	MH071062	MH070809	Chen et al. (2019)
*S. involucrata*	China, Xinjiang, Urumqi; WYJ20160725 (163)	43°6'30.49", 86°50'31.92"	3564	MH003736	MH070900	MH071026	MH070773	Chen et al. (2019)
*S. involucrata*	China, Xinjiang, Urumqi; WYJ20160725 (165)	43°6'30.49", 86°50'31.92"	3564	MH003737	MH070901	MH071027	MH070774	Chen et al. (2019)
*S. involucrata*	China, Xinjiang, Tekesi; WYJ201308184 (24)	43°5'56.94", 86°50'31.92"	3678	MH003738	MH0070902	MH071028	MH070775	Chen et al. (2019)
*Jurinea multiflora*	China, Xinjiang, Tuoli; WYJ201308102 (377)	45°44'8.3", 83°8'49.63"	1753	MH003704	MH070869	MH070995	MH070742	Chen et al. (2019)

Total genomic DNA was extracted from silica gel-dried leaf materials following the CTAB method (Doyle and Doyle 1987). The PCR reaction was performed in a 50 µl volume, containing approximately 200 ng DNA, 1.5 mM MgCl_2_, 0.2 mM dNTP, 1 µM of each primer and 0.75 units of Taq DNA polymerase. Initial template denaturation was programmed at 94 °C for 4 min and then followed by 30 cycles of 94 °C for 1 min, annealing at 50–56 °C for 1 min and extension at 72 °C for 1 min, with a final extension step of 72 °C for 7 min. Markers used for the amplification and sequencing are listed in Table 2. PCR products were sent to ZanaaSPX, Mongolia (www.hangal.mn) for commercial sequencing. Sequences were aligned using MEGA 7 (Kumar et al. 2016), with the default settings and manual adjustments were made using SnapGene Viewer 4.2.6. Sequences were edited manually using SnapGene Sequence Alignment Editor (GSL Biotech LLC). Ambiguous nucleotide bases were corrected using the corresponding base of the sequence that was obtained by the reverse primer. Multiple sequences were aligned using ClustalW with its default parameters (Thompson et al. 1994) and consensus sequences were created for each species. For the combined dataset, the genetic divergences were calculated using DNASP v.6 (Julio et al. 2017) and used to determine whether a barcoding gap was present. The DNA sequences generated in this study have been deposited in GenBank (Table 1).

**Table 2.** List of the markers used for the DNA barcoding and phylogenetic analysis.

Fragment	Marker	Sequence 5’ → 3’	T_a_	Reference
ITS	*ITS4*	TCCTCCGCTTATTGATATGC	50 °C	White et al. (1990)
	*ITS5A*	CCTTATCATTTAGAGGAAGG		
*rbcL*	*rbcL_f*	ATGTCACCACAAACAGAGAC	56 °C	Chase et al. (1993)
	*rbcL_r*	CTTCTGCTACAAATAAGAAT		
*trnK*	*trnK(UUU)*	TTAAAAGCCGAGTACTCTACC	50 °C	Sang et al. (1997)
	*rps16*	AAAGTGGGTTTTTATGATCC		
*trnH*-*psbA*	*psbA*	GTTATGCATGAACGTAATGCTC	56 °C	Olmstead et al. (1992)
	*trnH*	CGCGCATGGTGGATTCACAATCC		

The phylogenetic analyses were conducted using Bayesian Inference (BI), Maximum Likelihood (ML) and Maximum Parsimony (MP). For BI analysis, the best close fit model of evolution for each partition neighbour joining (NJ) tree was estimated using MEGA 7 (Kumar et al. 2016). Posterior probability was determined by Markov Chain Monte Carlo sampling (MCMC) with the programme MrBayes v. 3.2.6 (Huelsenberk and Ronquist 2001; Ronquist and Huelsenberk 2003), as implemented in Geneious v. 10.2.2 (Kearse et al. 2012), using the estimated models of evolution. For each dataset, four simulation Markov chains were run for 1 million generations and trees were sampled every 100^th^ generation. The ML analysis was performed using RAxML v. 8.2.11 (Stamatakis 2006, 2014) as implemented in Geneious v. 10.2.2 (Kearse et al. 2012), using the GTRGAMMA model with rapid bootstrapping and a search for the best-scoring ML tree algorithm, including 1,000 bootstrap replicates. The MP analyses were performed with MEGA 7 (Kumar et al. 2016), using tree-bisection-reconnection (TBR) as the branch-swapping algorithm. The robustness of the tree was evaluated using 1,000 bootstrap replication indices and the consistency index, retention index and composite index were calculated.

## Results

We discovered *S. bogedaensis* from Baitag Bogd Mt in the DzG region of Mongolia. This species is newly documented in the Mongolian flora. Detailed data on morphological and genetic identification, geographical distribution and conservation status of the *S. bogedaensis* are provided below.

### New record

#### Saussurea bogedaensis

Taxon classificationPlantae

Yu J.Wang & J.Chen, PloS ONE 13(7): e0199416 (12) (2018)

91F2AB3A-C282-5ED2-89CF-FD4BDD7DA7B3

Figs 1 , 3

##### Morphological identification.

**Figure 1.**
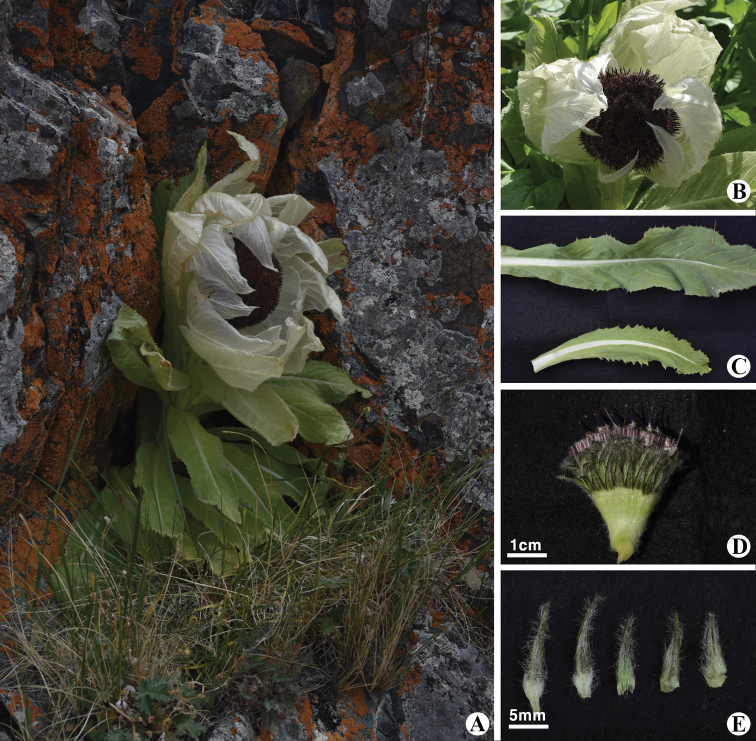
*Saussurea bogedaensis* in Buduun Khargait river, Baitag Bogd Mt, Uyench sum, Khovd Province, Mongolia. **A** general habit in Baitag Bogd Mt, DzG region **B** fruiting **C** leaves **D** pappus **E** phyllaries. Photos: 28 July 2019, Sh. Baasanmunkh.

**Figure 2.**
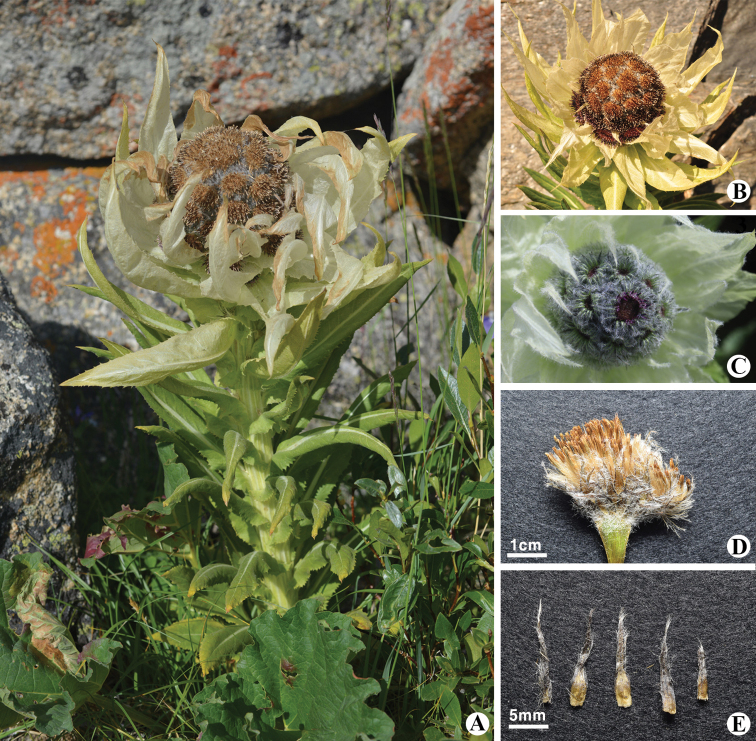
*Saussurea orgaadayi* in Khukh Nuur, Munkhkhairkhan sum, Khovd Province, Mongolia **A** general habit in Munkhkhairkhan Mt, MA region **B** fruiting **C** flowering **D** pappus **E** phyllaries. Photos: 29 July 2016, B. Oyuntsetseg (**A, C**) Sh. Baasanmunkh (**B, D, E**).

**Figure 3.**
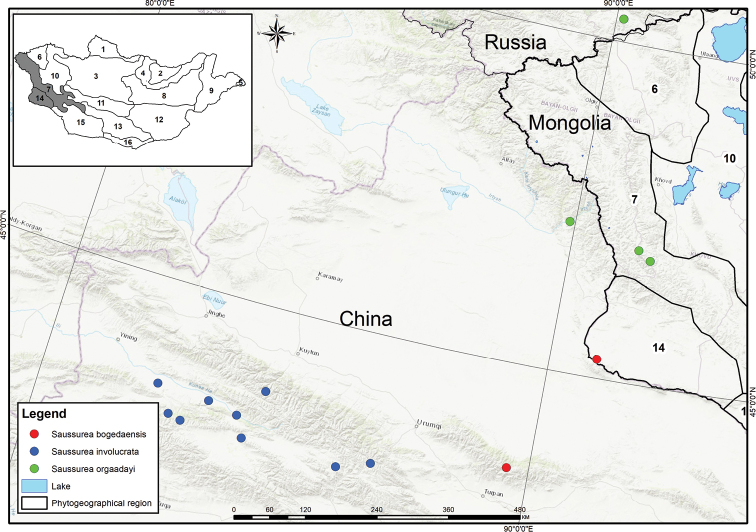
Distribution of *S. bogedaensis* (red dots), *S. involucrata* (blue dots) and *S. orgaadayi* (green dots) in Mongolia, Russia and Chinese Tien-Shan Mts based on field surveys and herbarium materials as well as specimens from China based on the publications of Chen and Wang (2018) and Chen et al. (2019). Region numbers on the Mongolian map are phytogeographical regions according to Grubov (1982): 6 – Khovd, 7 – Mongolian Altai, 10 – Depression of Great Lakes and 14 – Dzungarian Gobi.

*Saussurea bogedaensis* (Fig. 1) was recently discovered on Bogeda Mt in Xinjiang, China by Chen and Wang (2018) (Fig. 3). This species is very similar to *S. involucrata* and *S. orgaadayi* (Fig. 2), but several morphological characteristics of the bracts, involucres and phyllaries differentiate them (Chen and Wang 2018). In particular, *S. bogedaensis* differs by having elliptic, apically obtuse stem leaves (Fig. 1C) vs. lanceolate, long-acuminate stem leaves in *S. orgaadayi* (Fig. 2A); dirty white pappus colour (Fig. 1D) vs. straw-coloured pappi in *S. orgaadayi* (Fig. 2D); densely pubescent phyllaries (Fig. 1E) vs. glabrous phyllaries in *S. involucrata*; and campanulate involucres in *S. bogedaensis* vs. hemispherical involucres in *S. involucrata*.

##### Genetic identification.

**Figure 4.**
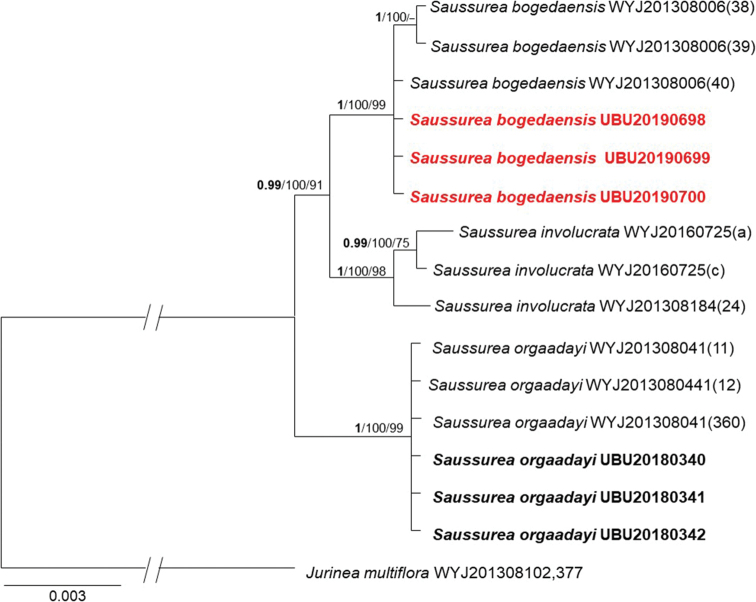
Phylogenetic tree based on concatenated sequence alignments of nrDNA (ITS) and cpDNA (*trnK*, *trnH-psbA*, and *rbcL*) regions. Bayesian Inference (BI) posterior probability support values above 90% (bold), Maximum Likelihood (ML) and Maximum Parsimony (MP) bootstrap support values above 70% are shown in the branches in the following order **BI**/ML/MP. The new samples of *S. bogedaensis* and *S. orgaadayi* originated from Mongolia are in red and black bolds, respectively.

The combined sequence dataset consisted of 15 samples, including the outgroup, *Jurinea multiflora*. The sequence dataset comprised 2,315 characteristics, of which 20 were parsimony-informative, 108 were variable and 2,169 were constant. The gene boundaries on the ITS – *trnK* – *trnH-psbA* – *rbcL* multi-locus alignment were as follows: ITS: 1–656, *trnK*: 657–1,284, *trnH-psbA*: 1,285–1,680 and *rbcL*: 1,681–2,315. The final ML optimisation likelihood of ML analysis was: Inl = -3650.7353. A single most parsimonious tree was generated by MP analysis with a tree length of 105 steps, consistency index: 1.0, retention index: 1.0 and composite index: 1.0. The BI phylogeny, including BI posterior probability values, as well as ML and MP bootstrap support values, are provided in Fig. 4.

**Figure 5.**
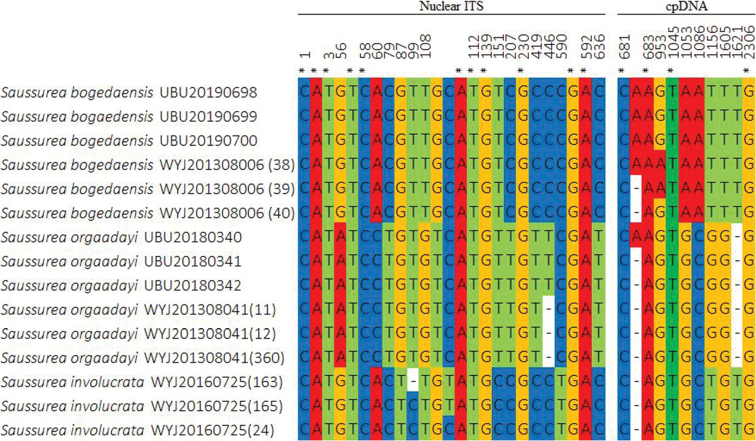
Multiple sequence alignment of combined nr DNA (ITS) and cpDNA (*trnK*, *trnH-psbA* and *rbcL*) sequences. ITS region shows more differences than cpDNA regions amongst those closely related species. (*) – no differences found between species.

Our genetic identification revealed a similar topology to that of Chen and Wang (2018) and confirms each distinct clade of *S. bogedaensis*, *S. involucrata* and *S. orgaadayi*, respectively (Fig. 4). Three individuals of newly-revealed *Saussurea* specimens from Baitag Bogd Mt formed one cluster with the Chinese *S. bogedaensis* with high support: BI/ML/MP = 1/100/99. Additionally, sequence divergence amongst the three species was 0–0.002% in our *S. bogedaensis* specimens, whereas there was 3.02% sequence divergence in *S. involucrata* and 2.04% sequence divergence in *S. orgaadayi*. Sequence alignment revealed that the Mongolian and Chinese *S. bogedaensis* share several specific nucleotide residues that are different from those of other *Saussurea* species (Fig. 5). The other three samples (Fig. 2) from Munkhkhairkhan Mt in the MA region clustered with *S. orgaadayi* from China (BI/ML/MP = 1/100/99). Therefore, our study proves that the *Saussurea* samples from the DzG and MA regions are *S. bogedaensis* (Fig. 1) and *S. orgaadayi* (Fig. 2), respectively. Our genetic results provide only the genetic differences between the three related species in the subg. Amphilaena and not a true phylogeny of all related *Saussurea* species.

##### General distribution and habitat.

Mongolia (Dzungarian Gobi, Baitag Bogd Mt) and China (Xinjiang, Bogeda Mt). In Mongolia, *S. bogedaensis* grows on high mountain rocky slopes, screes, boulders and river banks in the alpine belt at altitudes of 2400–3300 m a.s.l. This species is closely related to *S. involucrata* and *S. orgaadayi*. However, the three species are geographically isolated: *S. bogedaensis* occurs in the Dzungarian basin and the eastern Chinese Tien-Shan Mts and *S. involucrata* occurs in the Tien-Shan Mts (which cover parts of China and Central Asian states), whereas *S. orgaadayi* is present in the Altai Mts (which cover parts of China, Mongolia and Russia) (Fig. 3), according to Raab-Straube (2017) and Chen and Wang (2018).

##### Conservation status.

*Saussurea bogedaensis* is new to the Mongolian flora and occurs in the Baitag Bogd Mt in the DzG region. Individuals of the species were found in a few locations, namely in Baitag Bogd Mt and Altan Ovoo in the DzG region (Fig. 3). During our field surveys, we detected two different populations, which in total, accounted for fewer than 600 individuals in this region. This species is under threat, particularly owing to human interference and random cutting. Thus, *S. bogedaensis* has been assessed as Critically Endangered [CR C2a(i)] in Mongolia according to the IUCN Red List categories and criteria (IUCN 2019). This species was also evaluated as critically endangered in China (Chen and Wang 2018). *In situ* studies on the reproductive biology of *S. bogedaensis* are needed to more accurately assess the conservation status of this species in Mongolia.

##### Specimens examined (new record).

Mongolia. Dzungarian Gobi region: Khovd Province, Uyench sum, Baitag Bogd Mt, Buduun Khargait river, 45°13'14.52"N, 90°55'12.97"E, 2742 m a.s.l., 28 Jul 2019, *Sh. Baasanmunkh et al.*, *20190698*, *20190699*, *20190700* (UBU). The samples from this site were used for the molecular analysis confirming the identity of the Mongolian plants as *S. bogedaensis*.

## Discussion

*Saussurea bogedaensis*, *S. orgaadayi* and *S. involucrata* belong to the taxonomically complicated Saussurea subg. Amphilaena (Raab-Straube 2017). Despite their similar morphological characteristics and habitats, there are clear morphological differences, geographically isolated distributions and genetic identities that make these species recognisable with an in-depth investigation (Figs 1–5; Chen and Wang 2018; Chen et al. 2019). There are some distribution records of *S. involucrata* from the regions of Khovd and the Depression of Great Lake in Mongolia (Urgamal et al. 2014). Due to limited numbers of samples and surveyed areas of the MA and DzG regions in this study, data on Mongolian *S. involucrata* are still unclear. Hence, correct identification based on this study will provide an important basis for future studies on the taxonomic identity of Mongolian *S. involucrata*.

## Supplementary Material

XML Treatment for Saussurea bogedaensis
